# Preoperative Somatostatin Analogues in Patients with Newly-diagnosed Acromegaly: A Systematic Review and Meta-analysis of Comparative Studies

**DOI:** 10.1038/s41598-019-50639-6

**Published:** 2019-10-01

**Authors:** Chengxian Yang, Ge Li, Shenzhong Jiang, Xinjie Bao, Renzhi Wang

**Affiliations:** 1 Department of Neurosurgery, China Pituitary Disease Registry Center, Peking Union Medical College Hospital, Peking Union Medical College & Chinese Academy of Medical Sciences, Beijing, China; 2 Department of Endocrinology, Key Laboratory of Endocrinology of Ministry of Health, Peking Union Medical College Hospital, Peking Union Medical College & Chinese Academy of Medical Sciences, Beijing, China

**Keywords:** Pituitary diseases, CNS cancer, Surgical oncology

## Abstract

Biochemical remission after transsphenoidal surgery is still unsatisfied in acromegaly patients with macroadenomas, especially with invasive macroadenomas. Concerning the impact of preoperative somatostatin analogues (SSAs) on surgical outcomes, previous studies with limited cases reported conflicting results. To assess current evidence of preoperative medical treatment, we performed a systematic review and meta-analysis of comparative studies. A literature search was conducted in Pubmed, Embase, and the Cochrane Library. Five randomized controlled trials (RCT) and seven non-RCT comparative studies were included. These studies mainly focused on pituitary macroadenomas though a small number of microadenoma cases were included. For safety, preoperative SSAs were not associated with elevated risks of postoperative complications. With respect to efficacy, the short-term cure rate was improved by preoperative SSAs, but the long-term cure rate showed no significant improvement. For invasive macroadenomas, the short-term cure rate was also improved, but the long-term results were not evaluable in clinical practice because adjuvant therapy was generally required. In conclusion, preoperative SSAs are safe in patients with acromegaly, and the favorable impact on surgical results is restricted to the short-term cure rate in macroadenomas and invasive macroadenomas. Further well-designed RCTs to examine long-term results are awaited to update the finding of this meta-analysis.

## Introduction

Acromegaly is a rare disease characterized by the overproduction of growth hormone (GH), which is commonly secreted by a pituitary adenoma. The clinical presentations of acromegaly are systemic such as acral overgrowth, featured facial changes, sleep apnea, diabetes, hypertension, thyroid nodules, and colorectal polyps attributing to long-term exposure to high levels of GH and insulin-like factor-1 (IGF-1)^1^. Because of cardiovascular, respiratory, and metabolic comorbidities, patients with active acromegaly are associated with higher mortality^2–4^, though pituitary adenomas are commonly benign. Transsphenoidal surgery is the first-line treatment of choice for patients with acromegaly. Somatostatin analogues (SSAs) and radiotherapy are considered as adjuvant treatment. The goals of treatment consist of relieving clinical symptoms, controlling tumor growth, and achieving biochemical remission.

It is believed that the gross total resection of GH-secreting pituitary adenoma is associated with preoperative GH levels, tumor classification regarding invasiveness and size, and tumor consistency. In most patients, preoperative SSAs are proven to reduce GH and IGF-1 levels, stimulate tumor shrinkage, and soften tumor consistency. Theoretically, preoperative SSAs can promote gross total resection and biochemical remission. However, previous studies with relatively small sample sizes have shown conflicting results regarding the benefit of preoperative SSAs. Consequently, SSAs were not recommended for routine use in acromegaly before pituitary surgery^5^.

According to previous studies and meta-analyses, preoperative medical treatment was in favor of improving the short-term cure rate, which was thought to be exaggerated by a carryover effect. However, the impact of preoperative medical treatment on the long-term results has not been well demonstrated. Moreover, it remains uncertain whether preoperative medical treatment can benefit the invasive GH-secreting pituitary adenomas in terms of short- and long-term biochemical remission. Therefore, we performed a systematic review and meta-analysis of comparative studies to evaluate the impact of preoperative SSAs on the surgical results and postoperative complications based on the updated evidence.

## Materials and Methods

According to the Preferred Reporting Items for Systematic Reviews and Meta-analysis^6^, we prepared a prospective protocol of literature search, inclusion and exclusion criteria, outcomes of interest, and methods of statistical analysis.

### Strategy of literature search

We performed a literature search using databases of PubMed, Embase, and the Cochrane Library from inception to November 1, 2018. The following search terms in various combinations were searched: acromegaly, somatostatin analogue, octreotide, lanreotide, pasireotide, medical treatment, transsphenoidal, transcranial, and surgery. Only the articles written in the English language were included. Two researchers (C.Y. & S.J.) performed literature searches independently. If any discrepancy about the eligibility of an article occurred, a consensus was reached with the guidance of the senior authors (X.B. & R.W.).

### Inclusion and exclusion criteria

The literature search aimed to find articles that met the following inclusion criteria: (1) described a comparative study including patients treated with preoperative SSAs or direct surgery; (2) demonstrated the regimen of preoperative medical treatment in terms of dosage, frequency, and duration; (3) reported the number of patients and had at least five patients for each group; (4) reported at least one quantitative outcome of interest mentioned in the next section. Therefore, editorials, letters to editors, meeting abstracts, reviews, non-comparative studies, and case reports were excluded.

### Data extraction and outcome of interest

Two researchers (C.Y. & S.J.) extracted data of included studies independently. If there was any disagreement about the eligibility of data, a consensus was reached with the guidance of the senior authors (X.B. & R.W.). The outcomes of interest were short-term and long-term biochemical remission rates in all acromegaly patients (mainly macroadenomas), short-term biochemical remission rates in invasive macroadenomas, and postoperative complication rates. Biochemical remission is measured by one of following criteria: (1) nadir GH <1 ug/L during oral glucose tolerance test (OGTT) and normal levels of IGF-1 adjusted for age and sex; (2) nadir GH <0.4 ug/L during OGTT and normal levels of IGF-1 adjusted for age and sex^7^. Short-term biochemical remission is defined as follows: (1) conducted a biochemical assessment within six months after pituitary surgery; (2) satisfied cure criteria; (3) received no adjuvant therapy before the last biochemical assessment within six months. Long-term biochemical remission is similar as described previously except that the biochemical assessment is conducted after six months postoperatively. If there were several biochemical results during the short/long-term follow-up, the latest result was adopted. The rate was calculated to divide the number of events by the total number of evaluable patients in each group.

### Quality assessment and data analysis

The methodological quality of randomized controlled trials (RCT) was assessed using the Cochrane Risk of Bias Tool. The methodological quality of non-RCT comparative studies was assessed using the nine-star Newcastle-Ottawa scale (NOS)^8^. Non-RCT studies with more than six stars and RCTs were considered as high-quality studies in this analysis. The statistical analyses of pooled data were conducted using Review Manager, version 5.3.5 (The Nordic Cochrane Centre, The Cochrane Collaboration, 2014). The odds ratio to compare dichotomous variables was calculated using the method of the Mantel-Haenszel test. The random-effects model was performed if there was heterogeneity between selected studies; otherwise, the fixed-effects model was performed. Study heterogeneity was determined using the Cochrane Q and I2 statistics. Heterogeneity was considered significant when the p value from Cochrane Q was <0.1 or I^2^ >50%. Publication bias was assessed by visually inspecting the funnel plots^9^.

## Results

### Literature search

**Figure 1:**
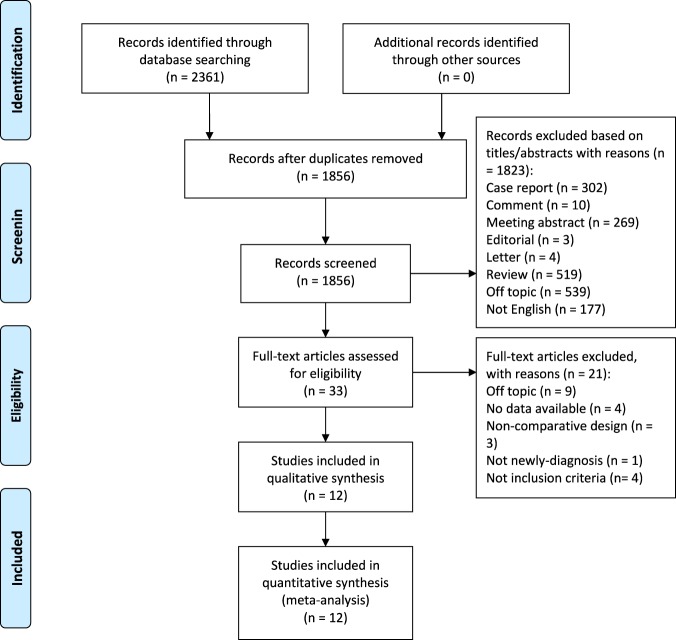
PRISMA flow diagram of literature search and study selection.

Our literature search strategy identified 2,361 articles, of which 505 duplicated articles were excluded. After the screening of titles and abstracts, 1,823 articles were excluded because of unrelated article types, irrelevant topics, and non-English writing. After reviewing 33 full-text articles in detail, a total of 21 articles were excluded because of no data available, unrelated topics, non-comparative design, and inappropriate clinical assessment. Finally, 12 eligible studies comprising 924 cases (445 cases for SSA; 479 cases for non-SSA) were included for the final meta-analysis^10–21^. The process of study selection is shown (Fig. 1).

### Characteristics of included studies

Among the 12 eligible studies, there were five RCTs, two prospective studies, and five retrospective studies. In non-RCT studies, tumor classification was the most common matching factor in a comparative design, followed by preoperative growth hormone levels. As for tumor size, the study subjects were pituitary macroadenomas alone in six studies and a mixture of micro- and macroadenomas in six studies. The fraction of the included microadenomas was so small that it was negligible. Short-acting SSAs were daily used in five studies before 2008, while long-acting SSAs were used every one to four weeks in seven studies. Patients received the transsphenoidal surgery alone in 11 studies, and the remaining study also adopted the transcranial procedure in selected patients. The characteristic of the included studies are shown (Table 1).

**Table 1:** Characteristics of the included studies in this meta-analysis.

Author	Year	Study design	Patients No. SSA/Non-SSA	Matching factor	Approach	Tumor size SSA/Non-SSA	Remission criteria	SSA regimen			Follow-up
								Dosage	Frequency	Duration	
Stevenaert	1996	R	SSA: 64 Non-SSA: 108	NA	TS	Micro: 33/31 (*) Macro: 31/77 (*)	1	Octreotide 100–500 ug	Tid	Range: 3 w-39 mo	Mean, 6.4 yr Range, 0.5–24 yr
Biermasz	1999	P	SSA: 19 Non-SSA: 19	1, 2	TS	Micro:5/5 Macro:14/14	1	Octreotide 150–1500 ug	Qd	Mean: 5 mo Range: 1–17 mo	SSA: 5.7 ± 0.5 yr Non-SSA: 4 ± 0.6 yr
Kristof	1999	P	SSA: 11 Non-SSA: 13	Ra	TS	Macro: 11/13	1	Octreotide 50–300 ug	Tid	Mean: 16.5 w	NA
Abe	2001	R	SSA: 71 Non-SSA: 53	1	TS	Micro: 7/14 Macro: 64/39	1	Octreotide 100–1500 ug	Qd	Mean: NA Range: 3–21 mo	SSA: 51.7 ± 1.4 mo Non-SSA: 49.3 ± 2.9 mo
Plöckinger	2005	R	SSA: 24 Non-SSA: 24	1, 2	TS	Macro: 24/24	1	Octreotide 100–500 ug	Tid	Range: 3–6 mo	SSA: > 4 yr Non-SSA: > 10 yr
Carlsen	2008	RCT	SSA: 31 Non-SSA: 30	Ra	TS	Micro: 5/5 Macro: 26/25	1	Octreotide 20 mg	Every 28 d	6 mo	SSA: > 3 mo Non-SSA: > 3 mo
Mao	2010	RCT	SSA: 49 Non-SSA: 49	Ra	TS	Macro: 49/49	1	Lanreotide 30 mg	Every 1–2 w	4 mo	SSA: > 4 mo Non-SSA: > 4 mo
Shen	2010	RCT	SSA: 19 Non-SSA: 20	Ra	TS	Macro: 19/20	1	Octreotide 20 mg	Every 28 d	3 mo	SSA: > 6 mo Non-SSA: > 6 mo
Li	2012	RCT	SSA: 24 Non-SSA: 25	Ra	TS	Macro: 24/25	1	Lanreotide 30 mg	Every 1–2 w	3 mo	SSA: > 3 mo Non-SSA: > 3 mo
Fougner	2014	RCT	SSA: 31 Non-SSA: 30	Ra	TS	Micro: 5/5 Macro: 26/25	1	Octreotide 20 mg	Every 28 d	6 mo	SSA: > 5 yr Non-SSA: > 5 yr
Abarel	2018	R	SSA: 64 Non-SSA: 46	NA	TS	Micro: 12/8 Macro: 42/33 (reported in 95 patients)	2	Octreotide 10–30 mg/Lanreotide 60–90 mg	Every month	Mean: 6.4 mo Range: 3–18 mo	SSA: 46.5 ± 35.2 mo Non-SSA: 57.8 ± 39.9 mo
Lv	2018	R	SSA: 38 Non-SSA: 62	NA	TS, TC	Macro: 38/62	1	Octreotide 20 mg/Lanreotide 30 mg	Every 4/2 w	Median 3 m range:1–13 m	SSA: > 6 mo Non-SSA: > 6 mo

Study design: R = retrospective study; P = prospective study; RCT = randomized controlled trial.

Approach: TS = transsphenoidal; TC = transcranial.

Matching factor: NA = not available; 1 = tumor classification; 2 = preoperative GH; Ra = randomized.

Tumor size: Macro = macroadenoma (>1 cm); Micro = microadenoma (<1 cm).

Tumor size (*): Macro = macroadenoma (>1.5 cm); Micro = microadenoma (<1.5 cm).

Remission criteria: 1 = nadir GH <1 ug/L during OGTT and normal levels of IGF-1; 2 = nadir GH <0.4 ug/L during OGTT and normal levels of IGF-1.

### Quality assessment of included studies

The included studies were all high-quality after careful assessment because they were either RCTs or Non-RCT studies with more than six stars. Among the five RCTs, randomization methods were reported in two studies, and allocation concealment was provided in one study (Table 2). Though the clinical outcome assessment of participants was not blinded, the judgment of biochemical remission and postoperative complications was still objective. Therefore, we considered the risk of detection bias as low without blinding of outcome assessment and labeled a star in the part of outcome assessment in NOS. For the seven non-RCT studies, the scores of NOS ranged from seven to eight, indicating high quality (Table 3).

**Table 2:** Summary of the risk of bias for each included study using the Cochrane Risk of Bias Tool for Randomized Controlled Trials.

Study	Random sequence generation (selection bias)	Allocation concealment (selection bias)	Blinding of participants and personnel (performance bias)	Blinding of outcome assessment (detection bias)	Incomplete outcome data (attrition bias)	Selective reporting (reporting bias)	Other bias
Carlsen	Unclear	Unclear	Low	Low	Low	Low	Low
Mao	Unclear	Unclear	Low	Low	Low	Low	Low
Shen	Unclear	Unclear	Low	Low	Low	Low	Low
Li	Low	Unclear	Low	Low	Low	Low	Low
Fougner	Low	Low	Low	Low	Low	Low	Low

**Table 3:** Summary of the risk of bias for each included study using the Newcastle-Ottawa scale for non-RCT studies.

Study	Selection				Comparability	Outcome			Total score
	Representativeness of the exposed cohort	Selection of the non exposed cohort	Ascertainment of exposure	Demonstration that outcome of interest was not present at start of study	Comparability of cohorts based on the design or analysis	Assessment of outcome	Was follow-up long enough for outcomes to occur	Adequacy of follow up of cohorts	
Stevenaert	1	1	1	1	0	1	1	1	7
Biermasz	1	1	1	1	1	1	1	1	8
Kristof	1	1	1	1	0	1	1	1	7
Abe	1	1	1	1	1	1	1	1	8
Plöckinger	1	1	1	1	1	1	1	1	8
Abarel	1	1	1	1	0	1	1	1	7
Lv	1	1	1	1	0	1	1	1	7

### Meta-analysis of short-term biochemical remission

**Figure 2:**
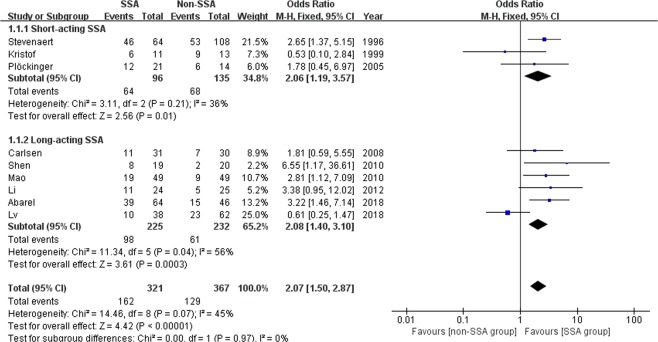
Forest plot of odds ratios with corresponding 95% CIs of studies on short-term biochemical remission in general acromegalics (mainly with macroadenomas) by medicine types.

Nine studies with a total of 688 patients mainly with macroadenomas evaluated the biochemical control within six months after pituitary surgery according to the widely-accepted remission criteria^10,12,14–18,20,21^. The meta-analysis was performed using the fix-effects model. The rate of short-term biochemical remission was 162/321 (50.5%) in the SSA group and 129/367 (35.1%) in the non-SSA group. The pooled data showed a significantly higher short-term cure rate in the SSA group than that in the non-SSA group (OR 2.07, 95% CI 1.50–2.87, p < 0.00001) (Fig. 2). The I^2^ statistic was 45%, demonstrating no significant heterogeneity among the included studies. Further, the ten studies were divided into two groups by short- (n = 3) or long-acting (n = 6) SSAs. A subgroup analysis was performed revealing the same result, which was in favor of preoperative medical treatment (OR 2.06, 95% CI 1.19–3.57, p = 0.01 and OR 2.08, 95% CI 1.40–3.10, p = 0.0003, respectively).

### Meta-analysis of long-term biochemical remission

**Figure 3:**
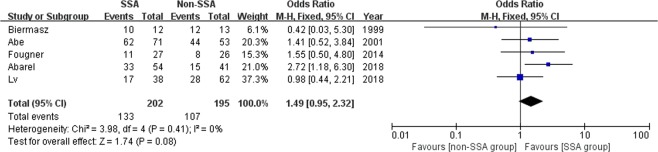
Forest plot of odds ratios with corresponding 95% CIs of studies on long-term biochemical remission in general acromegalics (mainly with macroadenomas).

Five studies with a total of 397 patients mainly with macroadenomas evaluated the biochemical control after over six months postoperatively according to the widely-accepted remission criteria^11,13,19–21^. The meta-analysis was performed using the fix-effects model. The rate of long-term biochemical remission was 133/202 (65.8%) in the SSA group and 107/195 (54.9%) in the non-SSA group. The pooled data showed no significant difference regarding the long-term cure rate between groups (OR 1.49, 95% CI 0.95–2.32, p = 0.08) (Fig. 3). The I^2^ statistic was 0%, demonstrating no significant heterogeneity among the included studies.

### Meta-analysis of biochemical remission in invasive macroadenomas

**Figure 4:**
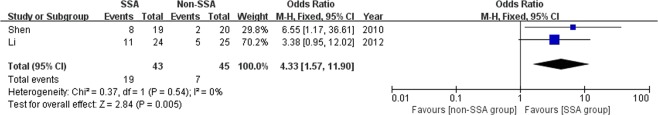
Forest plot of odds ratios with corresponding 95% CIs of studies on short-term biochemical remission in invasive macroadenomas.

As for invasive macroadenomas, two studies with a total of 88 patients evaluated the biochemical control within six months after pituitary surgery according to the widely-accepted remission criteria^17,18^. The meta-analysis was performed using the fix-effects model. The rate of short-term biochemical remission was 19/43 (44.2%) in the SSA group and 7/45 (15.6%) in the non-SSA group. The pooled data showed a significantly higher short-term cure rate in the SSA group than that in the non-SSA group (OR 4.33, 95% CI 1.57–11.90, p = 0.005) (Fig. 4). The I^2^ statistic was 0%, demonstrating no significant heterogeneity among the included studies. There was no publication for data synthesis about the long-term effect because adjuvant treatment was required in these cases, making it impossible to evaluate the real impact of preoperative medical treatment.

### Meta-analysis of postoperative complications

**Figure 5:**
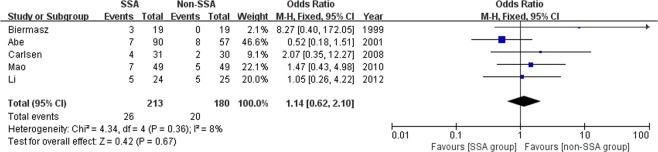
Forest plot of odds ratios with corresponding 95% CIs of studies on postoperative complications in general acromegalics (mainly with macroadenomas).

Five studies with a total of 393 patients evaluated the occurrence of postoperative complications^11,13,15,16,18^. The meta-analysis was performed using the fix-effects model. The rate of postoperative complications was 26/213 (12.2%) in the SSA group and 20/180 (11.1%) in the non-SSA group. The pooled data showed no significant difference regarding postoperative complications between groups (OR 1.14, 95% CI 0.62–2.10, p = 0.67) (Fig. 5). The I^2^ statistic was 8%, demonstrating no significant heterogeneity among the included studies.

### Publication bias

**Figure 6:**
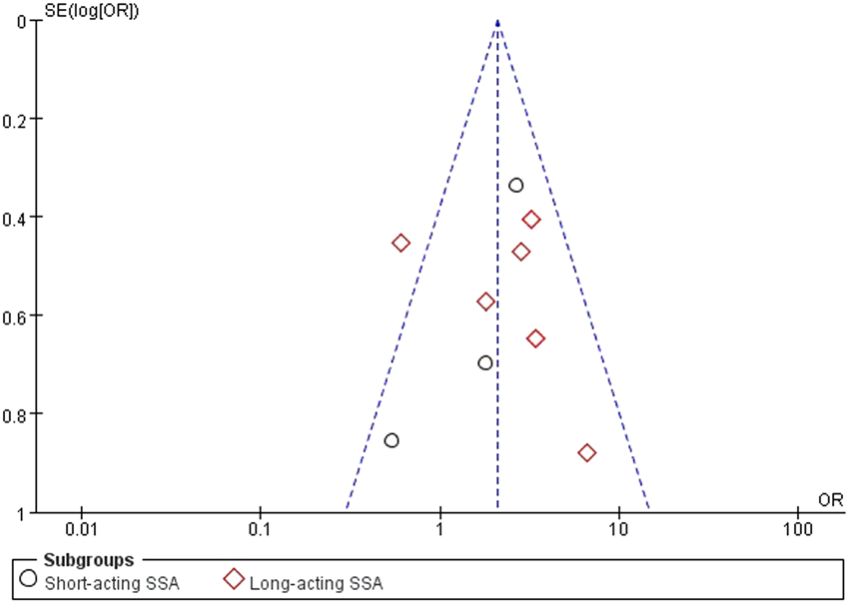
Funnel plot showing the symmetrical distribution of studies, indicating the absence of publication bias in studies on short-term biochemical remission in general acromegalics (mainly with macroadenomas).

**Figure 7:**
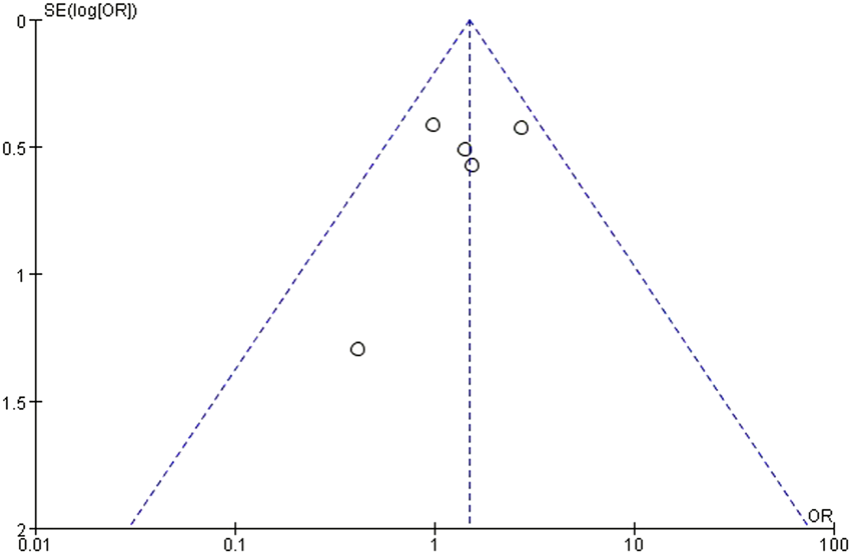
Funnel plot showing the symmetrical distribution of studies, indicating the absence of publication bias in studies on long-term biochemical remission in general acromegalics (mainly with macroadenomas).

**Figure 8:**
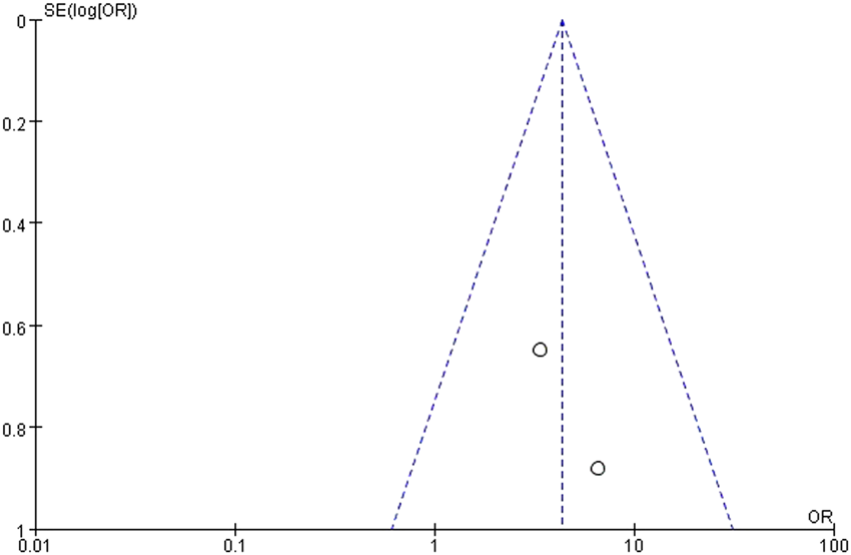
Funnel plot showing the symmetrical distribution of studies, indicating the absence of publication bias in studies on short-term biochemical remission in invasive macroadenomas.

**Figure 9:**
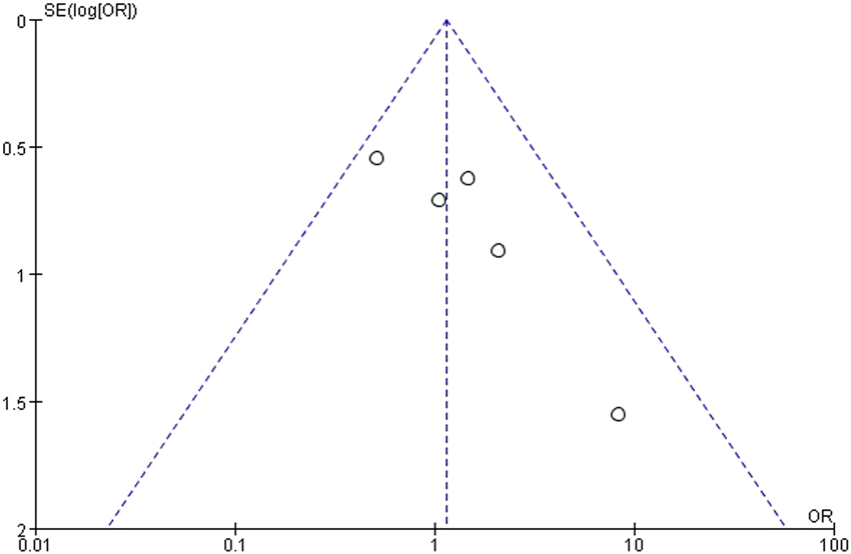
Funnel plot showing the symmetrical distribution of studies, indicating the absence of publication bias in studies on postoperative complications in general acromegalics (mainly with macroadenomas).

The funnel plots for all outcomes of interest were overall symmetrical. In each funnel plot, major studies were displayed within the 95% CI, with an even distribution around the vertical, suggesting no obvious publication bias (Figs 6–9).

## Discussion

There is a great debate about the place of preoperative SSAs in acromegaly treatment. Three meta-analyses have been published to summarize the conflicting results of previous studies. Francisco *et al*.^22^ included six controlled studies into a meta-analysis, detecting no significant difference in the cure rate between pretreatment and direct surgery groups (OR 1.62, 95% CI 0.93–2.82). However, when three RCTs were selectively analyzed, a significant effect was revealed to favor the impact of pretreatment on surgical results (OR 3.62, 95% CI 1.88–6.96). However, Francisco *et al*.^22^ neither considered the relation between tumor features and surgical difficulties nor distinguished the short- and long- term cure rate. Nunes *et al*.^23^ performed a meta-analysis based on four RCTs with a total of 248 cases. The result showed that preoperative medical treatment increased the three-month postoperative biochemical remission rate in GH-secreting pituitary macroadenomas (RR 2.15, 95% CI 1.39–3.33). Nevertheless, the three-month postoperative endocrine assessment was considered unpersuasive because of a carryover effect^5^. To explore more convincing evidence, Zhang *et al*.^24^ conducted another meta-analysis about the long-term effect of preoperative SSAs showing no significant difference (RR 1.03, 95% CI 0.86–1.24).

In our view, tumor invasiveness is the most critical factor to influence the impact of pretreatment. Several recent publications provided further clinical data. Therefore, it is necessary to perform an updated meta-analysis to refine the clinical evidence, with particular attention to invasive macroadenomas.

In this meta-analysis, we found that preoperative treatment of SSAs was associated with the elevated rate of short-term biochemical control in acromegaly, but no improvement was seen in the long-term cure rate. As for the acromegaly caused by invasive macroadenomas, which remained surgical challenges, preoperative treatment of SSAs could significantly improve the short-term cure rate as well. The data of long-term results were not available because radiotherapy, prolonged medical treatment, or both was commonly required in such cases with inoperable residual tumor. For safety, pretreatment exerted no impact on postoperative complications. Our results were consistent with guidelines and previous meta-analyses. Furthermore, this was the first study to report the value of preoperative SSAs in invasive macroadenomas achieving better short-term biochemical remission.

The effect of preoperative SSAs on surgical results was presumed to be multiple in terms of controlling GH, reducing tumor volume, softening tumor consistency, and lowering anesthetic risk. Theoretically speaking, these favorable results of pretreatment should improve the rate of gross total resection and biochemical control. Because the long-term result was unsatisfied, the favorable short-term result was questioned and thought to be due to a carryover effect. The results of unsatisfied long-term biochemical control were mainly from non-RCT studies except for the RCT study with a small sample conducted by Fougner *et al*.^19^. To elucidate the confusing phenomenon, we proposed the following potential reasons according to our experiences: (1) the included cases were a mixture of microadenomas and macroadenomas, whereas the pretreatment value was limited for microadenomas due to the high cure rate of approximately 90% by transsphenoidal surgery alone; (2) given that development of surgical techniques allows for more delicate resection of large and firm tumors, cavernous sinus invasion is the most significant predictor for surgical outcomes rather than preoperative hormone levels, tumor size and tumor consistency, which were considered as key therapeutic targets of preoperative SSAs. Thus, to clarify the impact of preoperative medical treatment more precisely, we recommend including patients with potentially-resectable invasive macroadenomas into a well-designed RCT.

The strengths of our meta-analysis are considered as follows. First, we made the most comprehensive literature search from three mainstream databases, including Pubmed, Embase, and Cochrane Library in order to find the updated clinical evidence. Second, before we performed the meta-analysis, we established a prospective protocol in terms of search strategies, data collection, dispute settlement, and statistical analysis, which was strictly followed in the whole procedure. Third, compared with previous studies, this meta-analysis included the largest number of cases from RCTs and non-RCTs, the qualities of which were relatively high after scale-based evaluation.

However, the present meta-analysis has the following limitations. First, the therapeutic regimen of preoperative somatostatin analogues varied significantly among the included studies. Second, retrospective and prospective cohort studies were included in the final analysis. Randomization is recognized to eliminate the baseline difference between groups. The included retrospective and prospective cohort studies were distinguished from RCTs for not adopting randomization in assigning patients. In three retrospective studies, methods to balance the baseline data were not even reported. Though one or two matching factors were stated in some studies, the inherent drawbacks of the non-RCT design might cause potential selection bias between groups. Third, considering the extensive perioperative use of SSAs, a considerable number of observational studies with negative results might be not reported. The publication bias should not be neglected. Fourth, original data did not show the biochemical control related to individual preoperative imaging features in size and invasiveness, reducing the application value of our results.

## Conclusion

Preoperative medical treatment leads to better short-term cure rates in the acromegalics, but its impact on the long-term results is unclear. In acromegalics with invasive macroadenomas, preoperative medical treatment also shows great efficacy in the short-term cure rate. As for safety, preoperative somatostatin analogues do not increase the risk of postoperative complications. Despite some limitations, the present meta-analysis provides the latest evidence regarding the safety and efficacy of preoperative SSAs.
